# BMP4 Signaling Is Able to Induce an Epithelial-Mesenchymal Transition-Like Phenotype in Barrett’s Esophagus and Esophageal Adenocarcinoma through Induction of SNAIL2

**DOI:** 10.1371/journal.pone.0155754

**Published:** 2016-05-18

**Authors:** Christine Kestens, Peter D. Siersema, G. Johan A. Offerhaus, Jantine W. P. M. van Baal

**Affiliations:** 1 Department of Gastroenterology and Hepatology, University Medical Center Utrecht, Utrecht, the Netherlands; 2 Department of Gastroenterology and Hepatology, Radboud University Medical Center, Nijmegen, The Netherlands; 3 Department of Pathology, University Medical Center Utrecht, Utrecht, the Netherlands; University of Colorado, Boulder, UNITED STATES

## Abstract

**Background:**

Bone morphogenetic protein 4 (BMP4) signaling is involved in the development of Barrett’s esophagus (BE), a precursor of esophageal adenocarcinoma (EAC). In various cancers, BMP4 has been found to induce epithelial-mesenchymal transition (EMT) but its function in the development of EAC is currently unclear.

**Aim:**

To investigate the expression of BMP4 and several members of the BMP4 pathway in EAC. Additionally, to determine the effect of BMP4 signaling in a human Barrett’s esophagus (BAR-T) and adenocarcinoma (OE33) cell line.

**Methods:**

Expression of BMP4, its downstream target ID2 and members of the BMP4 pathway were determined by Q-RT-PCR, immunohistochemistry and Western blot analysis using biopsy samples from EAC patients. BAR-T and OE33 cells were incubated with BMP4 or the BMP4 antagonist, Noggin, and cell viability and migration assays were performed. In addition, expression of factors associated with EMT (SNAIL2, CDH1, CDH2 and Vimentin) was evaluated by Q-RT-PCR and Western blot analysis.

**Results:**

Compared to squamous epithelium (SQ), BMP4 expression was significantly upregulated in EAC and BE. In addition, the expression of ID2 was significantly upregulated in EAC and BE compared to SQ. Western blot analysis confirmed our results, showing an upregulated expression of BMP4 and ID2 in both BE and EAC. In addition, more phosphorylation of SMAD1/5/8 was observed. BMP4 incubation inhibited cell viability, but induced cell migration in both BAR-T and OE33 cells. Upon BMP4 incubation, SNAIL2 expression was significantly upregulated in BAR-T and OE33 cells while CDH1 expression was significantly downregulated. These results were confirmed by Western blot analysis.

**Conclusion:**

Our results indicate active BMP4 signaling in BE and EAC and suggest that this results in an invasive phenotype by inducing an EMT-like response through upregulation of SNAIL2 and subsequent downregulation of CDH1.

## Introduction

Barrett’s esophagus (BE) is a premalignant condition in which the normal squamous epithelium (SQ) is replaced by intestinal type of epithelium containing goblet cells.[1] BE is a well-known risk factor for the development of esophageal adenocarcinoma (EAC). The incidence of EAC is steeply increasing in the Western world [2] and the prognosis remains poor with 5-year survival rates of 9% for metastatic and 25% for non-metastatic disease. [3] In the past decade many genes with an aberrant expression pattern during the development of BE and EAC have been reported. [4–6] However, understanding the cellular consequences of altered gene expression is far from complete.

Bone morphogenetic proteins (BMPs) belong to the transforming growth factor β family (TGF-β) and have multiple roles in cell proliferation, differentiation and migration. The BMP family consists of more than 20 members each having their own BMP-receptor affinity and regulating ligands. BMPs bind to transmembrane serine-threonine kinase receptors type 1 and 2 and activation of these receptors leads to phosphorylation of SMAD1, 5 and 8, which permits binding to SMAD4. This complex translocates to the nucleus and regulates gene transcription specific for the BMP pathway. Most of the functions of BMPs were thought to take place during embryogenesis, but recent studies have shown that they are aberrantly expressed in various malignancies and play a major role in cancer development. [7,8]

One of the BMPs, BMP4, is known to have an essential role in the development of BE. Milano *et al*. reported that the BMP4 pathway was activated in inflamed epithelium of the esophagus and in BE, in contrast to SQ were no active BMP4 signaling was found. [9,10] Zhou *et al*. reported that after bile and acid exposure, BMP4 expression was upregulated in human squamous epithelium cells. [11] Moreover, incubation of squamous cell cultures with BMP4 leads to a cytokeratin expression profile resembling that of BE. [9] This all suggests that in reflux esophagitis, the BMP4 pathway is activated which can lead to an intestinal type epithelium. [12] The molecular mechanism behind this metaplasic process is largely unknown; however, recent work of Mari *et al*. showed that binding of CDX2 and pSMAD 1/5/8 to the MUC2 promoter gene leads to the induction of MUC2 expression[13], which is one of the most characteristic intestinal markers highly expressed in BE. [10]

During the malignant progression from BE to EAC, oncogenes and tumor supressor genes are differently expressed leading to acquisition of invasive characteristics and eventually to metastasis. In the past, several studies have reported the ability of BMP4 to induce SNAIL2 expression. [14–20] SNAIL2 is one of the transcription factors regulating epithelial-mesenchymal transition (EMT). This transdifferentiation process results in cellular detachment from the basement membrane and controls cell motility. [21] Cells which have undergone EMT will express stromal keratins i.e. Vimentin. In cancer, cells most frequently de-differentiate rather than transdifferentiate showing an EMT like response. [22] A limited number of studies have focused on the role of EMT and/or an EMT-like response in relation to the malignant progression from BE to EAC. [23–25]

Until now, studies regarding the expression of BMP4 and the effect of BMP4 signaling in EAC are limited. We hypothesized that BMP4 could play a role in the progression from BE to EAC by inducing an EMT-like response. Therefore, we determined the expression of BMP4 and several members of the BMP4 pathway in EAC and investigated the effect of BMP4 signaling in a human Barrett’s esophagus (BAR-T) and an esophageal adenocarcinoma (OE33) cell line.

## Materials and Methods

### Biopsy specimens

Between September 2009 and May 2013, SQ, BE and EAC biopsy specimens were obtained from patients with BE associated EAC. Patient characteristics are shown in Table 1. Biopsies were collected during routine endoscopy and immediately frozen in liquid nitrogen and stored at -80°C. Pairwise-taken control biopsies were obtained to confirm the histological diagnosis. Patients were not treated with neoadjuvant chemotherapy and/or radiotherapy prior to biopsy sampling. All patients used proton pump inhibitor therapy. This study was approved by the Medical Ethics Committee of the University Medical Center Utrecht. All patients signed informed consent.

**Table 1:** Patient characteristics of biopsies used for Western blot, IHC and Q-RT-PCR analysis.

Number	Sex	Age (Years)	TNM stage	
EAC 1	M	59	T3N4	WB and IHC
EAC 2	M	49	T3N2	WB and Q-RT-PCR
EAC 3	M	73	T2N0	WB, IHC and Q-RT-PCR
EAC 4	M	65	T2N0	WB, IHC and Q-RT-PCR
EAC 5	M	80	T2N1	WB, IHC and Q-RT-PCR
EAC 6	M	42	T3N1	WB, IHC and Q-RT-PCR
EAC 7	M	81	T1N0	IHC
EAC 8	M	57	T1N1M0	IHC
EAC 9	M	68	T2N0M0	IHC
EAC 10	F	39	T3N0M0	IHC
EAC 11	M	67	T3N1	IHC
EAC 12	M	69	T3N1M1a	IHC
EAC 13	M	69	T3N0M0	IHC
EAC 14	M	72	T4N1M1a	IHC and Q-RT-PCR
EAC 15	M	61	T2N0	Q-RT-PCR
EAC 16	M	79	T3N2	Q-RT-PCR
EAC 17	M	70	T3N1M0	Q-RT-PCR
EAC 18	M	47	T3N1M1a	Q-RT-PCR

M = male, F = female, WB = Western blot, IHC = Immunohistochemistry, Q-RT-PCR = Quantitative Real Time PCR.

### RNA isolation and Quantitative Reverse Transcriptase Polymerase Chain Reaction (Q-RT-PCR)

Total RNA from biopsy specimens was extracted using RNA easy isolation kit, while total RNA from cell culture experiments was extracted using miRNeasy Mini kit (both Qiagen, Venlo, the Netherlands), according to the manufacturer’s instructions. RNA concentrations were measured using Nanodrop technology (Thermo Scientific, Erembodegem-Aalst, Belgium); all samples had an excellent 260/280 ratio. cDNA synthesis and Q-RT-PCR reactions were performed as previously described. [26] Primer sequences and annealing temperatures are shown in Table 2. Taqman assays (Applied Biosystems, Forster City, CA, USA) were used to measure the expression of BMPR1a (Hs01034913_g1), BMPR1b (Hs00176144_m1), BMPR2 (Hs00176148_m1) and SNAIL2 (Hs00950344_m1). Expression of the reference genes GAPDH (Hs4333764F) and B2M (Hs4333766F) were used for normalization.

**Table 2:** Primer sequences.

Target	Forward primer	Reverse primer	Annealing temperature (°C)
BMP4	TGAGCCTTTCCAGCAAGTTT	CTTCCCCGTCTCAGGTATCA	57.5
SMAD1	CACAAACATGATGGCGCCT	CATAGTAGACAATAGAGCACCAGTGTTT	58
SMAD4	TCCCGGACATTACTGGCCTGTTCA	GCGATCTCCTCCAGAAGGGTCCA	57.5
SMAD5	TTCTGGCTCAATCTGTCAACC	GGAGCCATCTGAGTAAGGAC	57.5
ID2	ACTCGCATCCCACTATTGTC	CGTCCATTCAACTTGTCCTCC	60
CDH1	CGAGAGCTACACGTTCACGG	GGGTGTCGAGGGAAAAATAGG	58
CDH2	TGGGAATCCGACGAATGG	TGCAGATCGGACCGGATACT	58
Vimentin	GGAAGCCGAAAACACCCTG	GAGACGCATTGTCAACATCCT	58

Primer sequences used for Q-RT-PCR with corresponding annealing temperature

Relative expression was calculated using the ΔC_t_ method. [27] All reactions were performed in duplicate. Data were expressed relative to SQ biopsies, control cells or cells incubated with Noggin.

### Western blot analysis

Protein samples were extracted using cell lysis buffer (Cell Signaling, Boston, MA, USA) containing 1% protease inhibitor (Sigma, St Louis, MO, USA). Cell lysates were centrifugated at maximum speed for 5 minutes at 4°C and the pellet was discarded. The lysates were diluted in protein sample buffer (100mM Tris pH 6.8, 2% β-mercaptoethanol, 4% SDS, 0.2% bromphenol blue, 20% glycerol) and incubated at 95°C for 5 min. Samples were loaded onto a 10% SDS-PAGE gel and subsequently transferred onto a PVDF membrane (Millipore, Amsterdam, the Netherlands). The blots were blocked with Odyssey Blocking buffer (Westburg, Leusden, the Netherlands) diluted 1:1 in Tris buffered saline (TBS) and subsequently washed with TBS. Blots were incubated 30–60 minutes at room temperature or overnight at 4°C with the primary antibody (Table 3) in Odyssey Blocking buffer diluted 1:1 in TBS supplemented with 0.1% Tween-20. After washing, blots were incubated with Alexa Fluor 680 conjugated secondary antibody (Invitrogen, Bleiswijk, the Netherlands) for 1 hour at room temperature. Visualization was performed using an Odyssey scanner (Westburg).

**Table 3:** Antibodies used for Western blot analysis.

Protein	Company	Dilution
BMP4	R&D Systems Minneapolis, Minnesota, USA	1:1000
BMPR1a (ALK-3)	R&D Systems Minneapolis, Minnesota, USA	1:500
BMPR1b (ALK-6)	R&D Systems Minneapolis, Minnesota, USA	1:400
BMPR2	R&D Systems Minneapolis, Minnesota, USA	1:1000
SMAD1	Cell Signaling Technology, Danvers, Massachusetts, USA	1:1000
SMAD4 (B-8)	Santa Cruz Biotechnology, Santa Cruz, California, USA	1:1000
pSMAD 1/5/8 (Ser463/465 Ser463/465 Ser426/428)	Cell Signaling Technology, Danvers, Massachusetts, USA	1:1000
ID2 (C-20)	Santa Cruz Biotechnology, Santa Cruz, California, USA	1:1000
SNAIL2 (C19G7)	Cell Signaling Technology, Danvers, Massachusetts, USA	1:1000
CDH1	BD Biosciences, San Jose, California, USA	1:2000
Actin (I-19)	Santa Cruz Biotechnology, Santa Cruz, California, USA	1:2000

### Immunohistochemistry

Four μm tissue sections were deparaffinized and blocked for endogeneous peroxidase activity by immersion in 0.3% H_2_O_2_ in methanol for 20 minutes. Antigen retrieval was performed in Tris/EDTA buffer (10 mM/1mM; pH 9.0) for 20 minutes at 100°C. After cooling for 10 minutes and washing in PBS pH 7.4, nonspecific binding sites were blocked with Serum Free Protein Block (Dako, Glostrup, Denmark) for 10 minutes followed by 1 hour incubation with SMAD4 antibody (Santa Cruz technology, Santa Cruz, CA, USA) at room temperature. Antibody binding was visualized using the BrightVision poly-HRP detection system (Immunologic, Duiven, the Netherlands) with 3,3-diamino-benzidine as chromogen. Sections were counterstained using hematoxylin, dehydrated and coverslipped using Pertex. Pancreas cancer tissue sections were used as negative control, showing no SMAD4 staining [28]. Control slides in which the primary antibody was omitted, were included. All sections were evaluated using a binocular microscope (Olympus BX 51, Zoeterwoude, the Netherlands).

### Cell culture

The human telomerase-immortalized non-neoplastic Barrett epithelium cell line, BAR-T [29] was kindly provided by Dr. R.F. Souza and cultured in Keratinocyte basal medium-2 (Lonza, Breda, the Netherlands), as described by Jaiswal *et al*. [29] Cell culture experiments were performed in collagen IV-coated wells (BD Biosciences, San Jose, CA, USA). The human esophageal adenocarcinoma cell line, OE33 (ECACC, Porton Down, Salisbury, United Kingdom) was cultured in RPMI-1640 with L-glutamine medium (Invitrogen) supplemented with 10% fetal bovine serum (FBS; Glico, Bleiswijk, the Netherlands) and 100 U/ml penicillin (Invitrogen) and 100 μg/ml streptomycin (Invitrogen). To evaluate the effect of BMP4 signaling, cells were seeded and after overnight attachment, the medium was replaced with serum reduced medium (0.2% FBS). Cells were incubated with 100 ng/ml recombinant human BMP4 or with 500 ng/ml Noggin Fc Chimera (both from R&D Systems, Minneapolis, MN, USA). All experiments were performed in duplicate and repeated at least 3 times.

### Cell viability

Cell viability was measured using MTT-assay (ATCC, Manassas, VA, USA), according to the manufacturer’s instructions. After 24 and 48 hours of incubation, colorimetric analysis was performed at 570 nm (reference 655 nm; Molecular Devices, Sunnyvale, CA, USA). All experiments were performed in duplicate and repeated at least 3 times.

### Cell migration

*In vitro* scratch assays were performed to evaluate cell migration; cells were seeded in a 12-well plate and after 24 hours scratched with a 10 μl pipette tip to create an artificial wound. Cells were washed twice with PBS to remove cellular debris and fresh medium was added. After 24 hours photographs of the scratches were taken. Images of the areas were taken using an EVOS microscope (Westburg).

To confirm our results from the scratch assays, Boyden chamber migration assays were performed. BAR-T cells suspended in serum free medium with BMP4 or Noggin were plated in the upper part of a 24-well, 8-μm pore, cell migration chamber (Cell biolabs inc, San Diego, CA, USA) according to the manufacturer’s protocol. Medium with 10% FBS was placed in the lower wells as chemotactic stimulus. Cells were allowed to migrate for 24 hours in a 37°C incubator with 5% CO_2_. Optical density of the dye was measured at 560 nm in a 96-well microtiter plate (Molecular Devices). All experiments were performed in duplicate and repeated at least 3 times.

### Statistical analysis

Data were analyzed using GraphPad Prism version 6.02 (San Diego, CA, USA). mRNA expression from biopsy specimens were presented as box plots, comparisons between different disease states were calculated using unpaired Student’s t-test or Mann-Withney U test when appropriate. The Mann-Withney U test was used to evaluate the effect of incubation with BMP4 or Noggin on ID2 expression in both BAR-T and OE33 cells. In all other cell culture experiments, data were expressed as mean ± standard deviation (SD) or standard error of the mean (SEM) and comparisons between groups were calculated using a Wilcoxon test. To exclude the effect of any active BMP4 signalling in control cells on mRNA expression, the results of cell culture experiments were compared to cells incubated with Noggin instead of control cells. A p-value of < 0.05 was considered statistically significant.

## Results

### Active BMP4 signaling in BE and EAC

**Fig 1:**
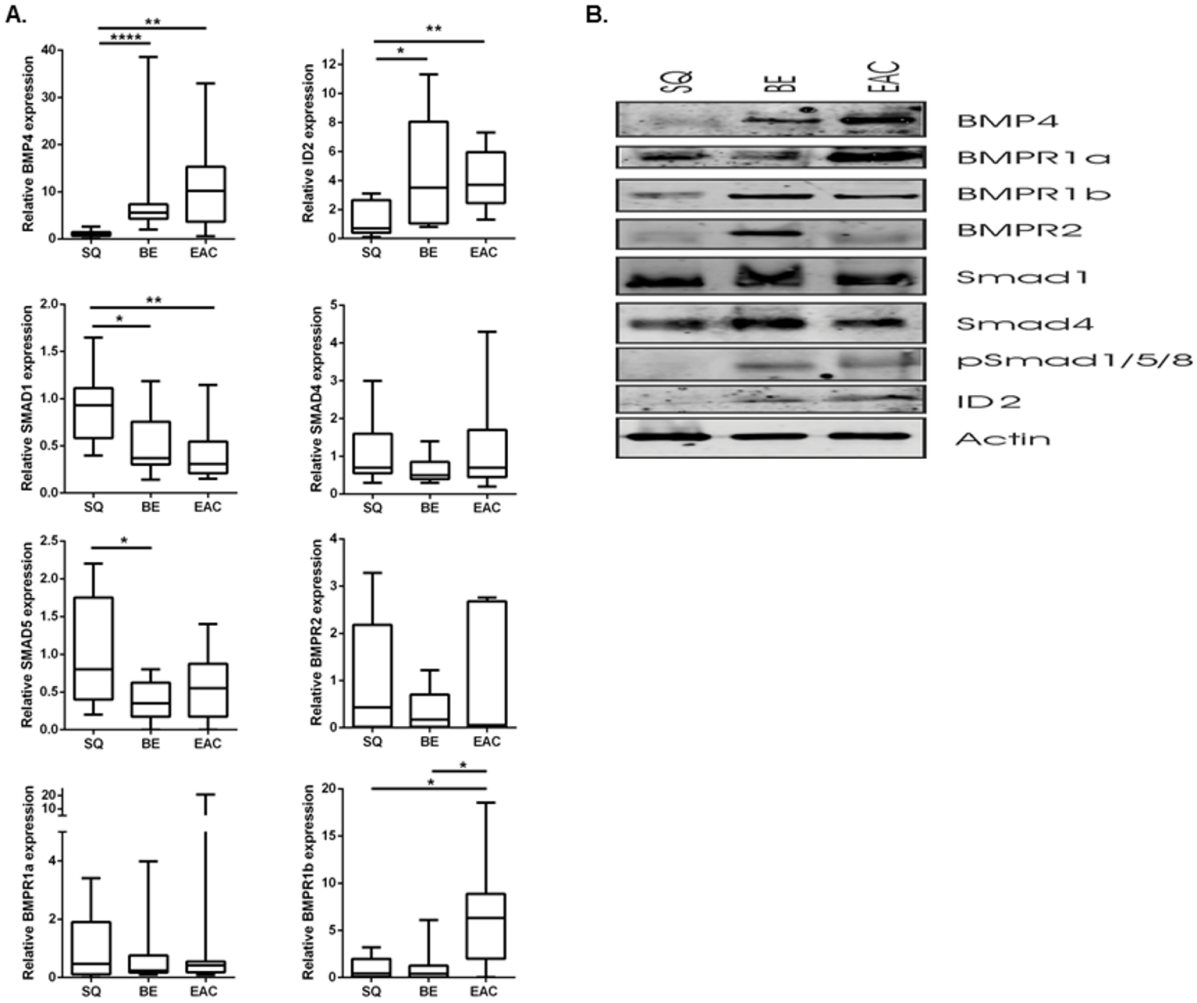
Expression of BMP4, BMP4 pathway associated molecules and the downstream target ID2. a. Q-RT-PCR was used to determine mRNA expression of BMP4, its downstream target, ID2, BMP4 associated receptors and SMAD molecules in SQ, BE and EAC biopsy specimens. B2M and GAPDH were used for normalization. Data are relative to the mean ΔCt of SQ biopsies and are expressed as box plots, representing the mean with the minimum and maximum values. *p<0.05, **p<0.01, ***p<0.001, ****p<0.0001. b. Western blot analysis in SQ, BE and EAC biopsy specimens showed BMP4, and ID2 expression and phosphorylation of SMAD1/5/8 in BE and EAC. Actin was used as loading control. Biopsy samples from 6 EAC patients were used. Representative pictures are shown. c. IHC showed nuclear and cytoplasmic expression of SMAD4 in 10 of 13 BE (arrowhead) and 11 out of 13 EAC tissue sections. EAC* represents a biopsy specimen with positive SMAD4 staining, EAC** represents a biopsy specimen with negative SMAD4 staining, stromal cells are SMAD4 positive and serve as internal control. Haematoxylin counterstain was used. Representative pictures are shown.

To determine whether there is active signaling in BE and EAC, expression of BMP4, molecules involved in the BMP4 signaling pathway and its downstream target, ID2, were determined in SQ, BE and EAC biopsy specimens by Q-RT-PCR (Fig 1a). BMP4 was 11.2-fold (p = 0.001) upregulated increased in EAC and 8.9-fold (p < 0.0001) upregulated in BE compared to SQ. The downstream target, ID2, was significantly upregulated in both EAC (3.2-fold, p = 0.002) and BE (3.5-fold, p = 0.02) compared to SQ. Of the BMP receptors, BMPR1b was significantly upregulated in EAC compared to BE and SQ (both p = 0.02). In contrast, SMAD1 was 2.2-fold (p = 0.008) downregulated in EAC and 1.8-fold (p = 0.02) downregulated in BE compared to SQ. SMAD5 was 2.6-fold (p = 0.04) downregulated in BE compared to SQ. BMP receptors BMPR1a, BMPR2 and SMAD4 were all expressed and were not found to be statistically different between the various groups (Fig 1a).

Next to mRNA expression, we determined protein expression levels by Western blot analysis. Upregulated expression of BMP4 and ID2 were observed in both BE and EAC (Fig 1b). In addition, more activation of pSMAD1/5/8 was observed in BE and EAC. In contrast, SQ did not show BMP4, and ID2 expression or pSMAD1/5/8 activation.

SMAD4 is often aberrantly expressed in malignancies and we therefore determined SMAD4 expression in EAC biopsy specimens using immunohistochemistry (Fig 1C). Eleven of 13 EAC specimens showed diffuse SMAD4 expression in both the nucleus and cytoplasm. The other 2 EAC specimens showed no SMAD4 expression. SMAD4 expression was present in 9 of 13 and 10 of 13, SQ and BE biopsy specimens respectively. Similar to EAC, BE specimens showed diffuse SMAD4 expression in both the nucleus and cytoplasm. In contrast, SQ specimens showed nuclear SMAD4 expression in the basal cells adjacent to the basement membrane, however no SMAD4 expression was observed in the more mature epithelial cells of the superficial layers (Fig 1C).

### In vitro BMP4 signaling showed a decrease in cell viability and an increase in migration in both a BE and EAC cell line

**Fig 2:**
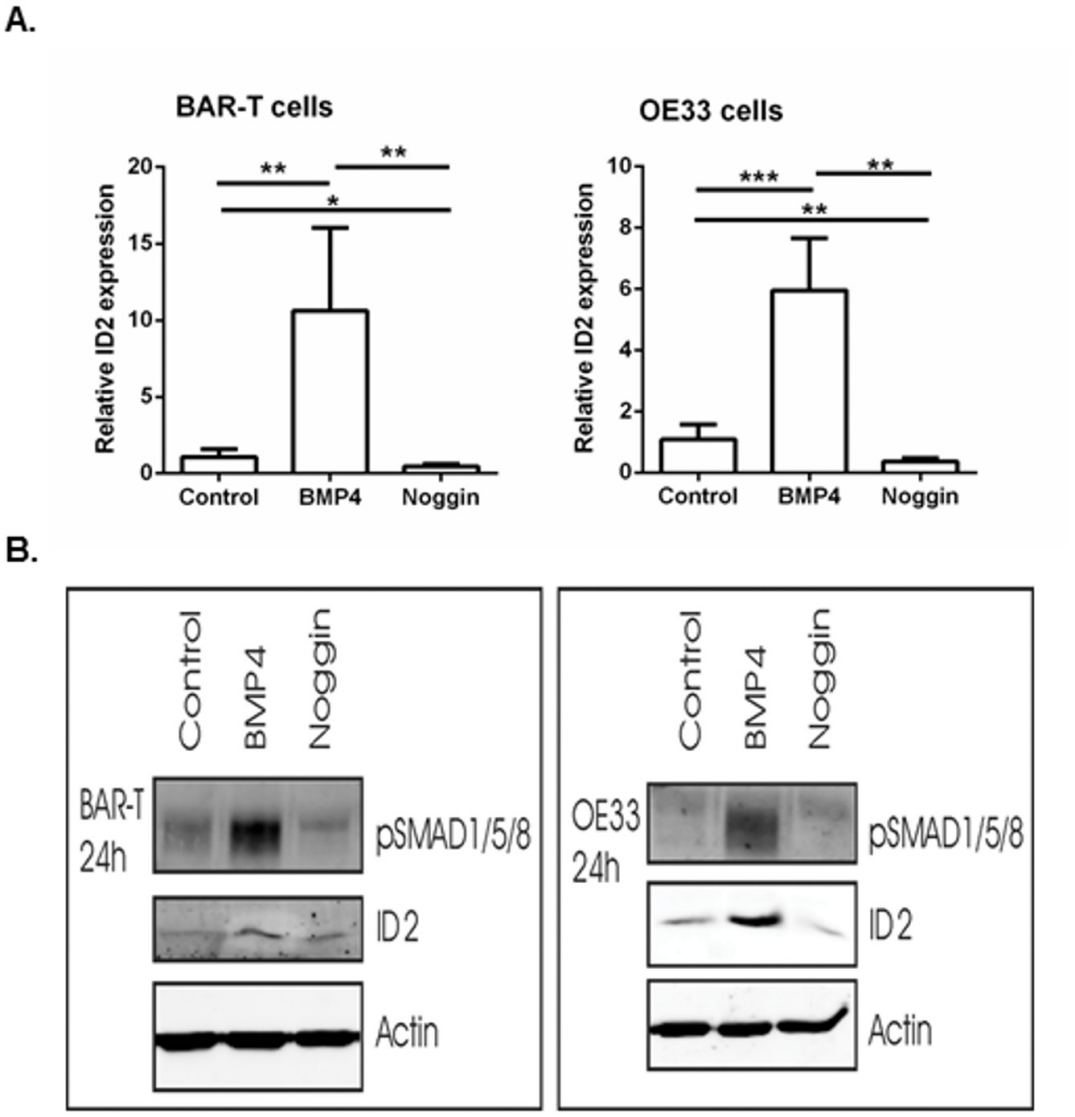
ID2 expression upon BMP4 or Noggin incubation. a. mRNA expression of the downstream target ID2. B2M was used for normalization. Data are relative to the mean ΔCt of control cells and are expressed as mean±SEM. *p<0.05, **p<0.01, ***p<0.001. b. Western blot analysis of BAR-T and OE33 cells incubated with BMP4 showed upregulated ID2 expression and more phosphorylation of SMAD1/5/8. Cells incubated with Noggin showed downregulated ID2 expression. Actin was used as loading control.

As a control, we first determined ID2 expression in BAR-T and OE33 cells upon BMP4 and Noggin incubation. Q-RT-PCR results showed a significantly upregulated ID2 expression in cells incubated with BMP4. A significant downregulation of ID2 expression was observed in cells incubated with Noggin compared to control cells. This effect was confirmed by Western blot analysis which also showed more phosphorylation of SMAD1/5/8 (Fig 2).

To explore the functional effect of BMP4 incubation in BAR-T and OE33 cells, cell viability and migration assays were performed. The results of the cell viability assays can be found in the supplementary data (S1 Fig).

**Fig 3:**
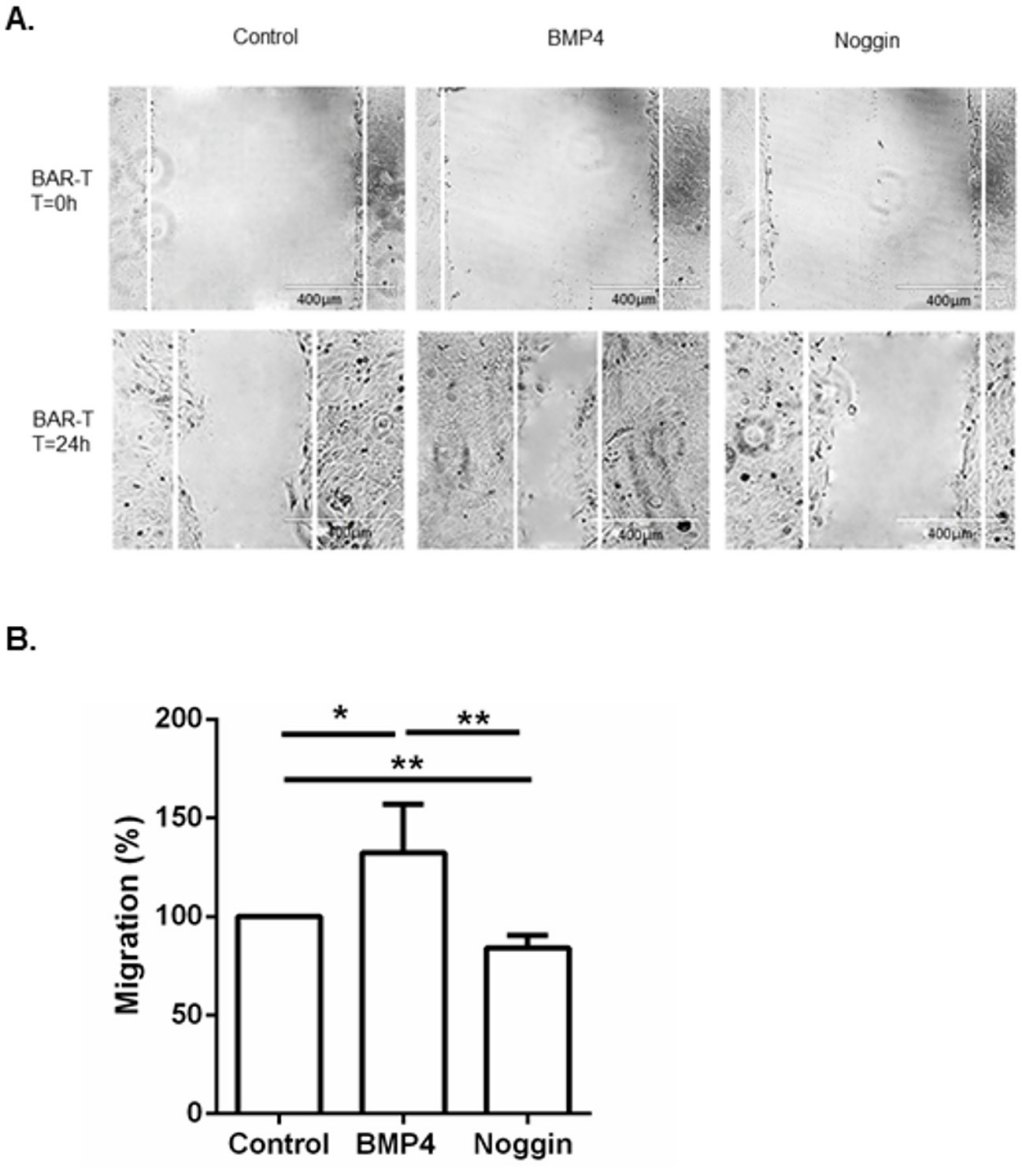
Migration assays of BAR-T and OE33 cells upon incubation with BMP4 or Noggin. a. *In vitro* scratch assay of BAR-T and OE33 cells. The rate of migration across the scratched area was monitored for 24 h. Representative images showed that the scratch induced cells incubated with BMP4 to migrate compared to control cells or cells incubated with Noggin. Images are representative of three independent experiments. b. Boyden chamber assay of BAR-T cells incubated with BMP4 or Noggin. Data are relative to control cells not incubated with BMP4 or Noggin and expressed as mean ±SD. * p<0.05, ** p<0.01.

Results from the scratch assays showed an increase in cell migration in both BAR-T and OE33 cells upon BMP4 incubation compared to cells incubated with Noggin and control cells (Fig 3a). These results were confirmed by a Boyden chamber assay for BAR-T cells; after 24 hours we observed a significant increase in cell migration in cells incubated with BMP4 compared to cells incubated with Noggin, 132% (±24.5) versus 84% (± 6.3; p = 0.006) respectively (Fig 3b).

### Upregulated SNAIL2 expression upon BMP4 incubation in BE and EAC cells

**Fig 4:**
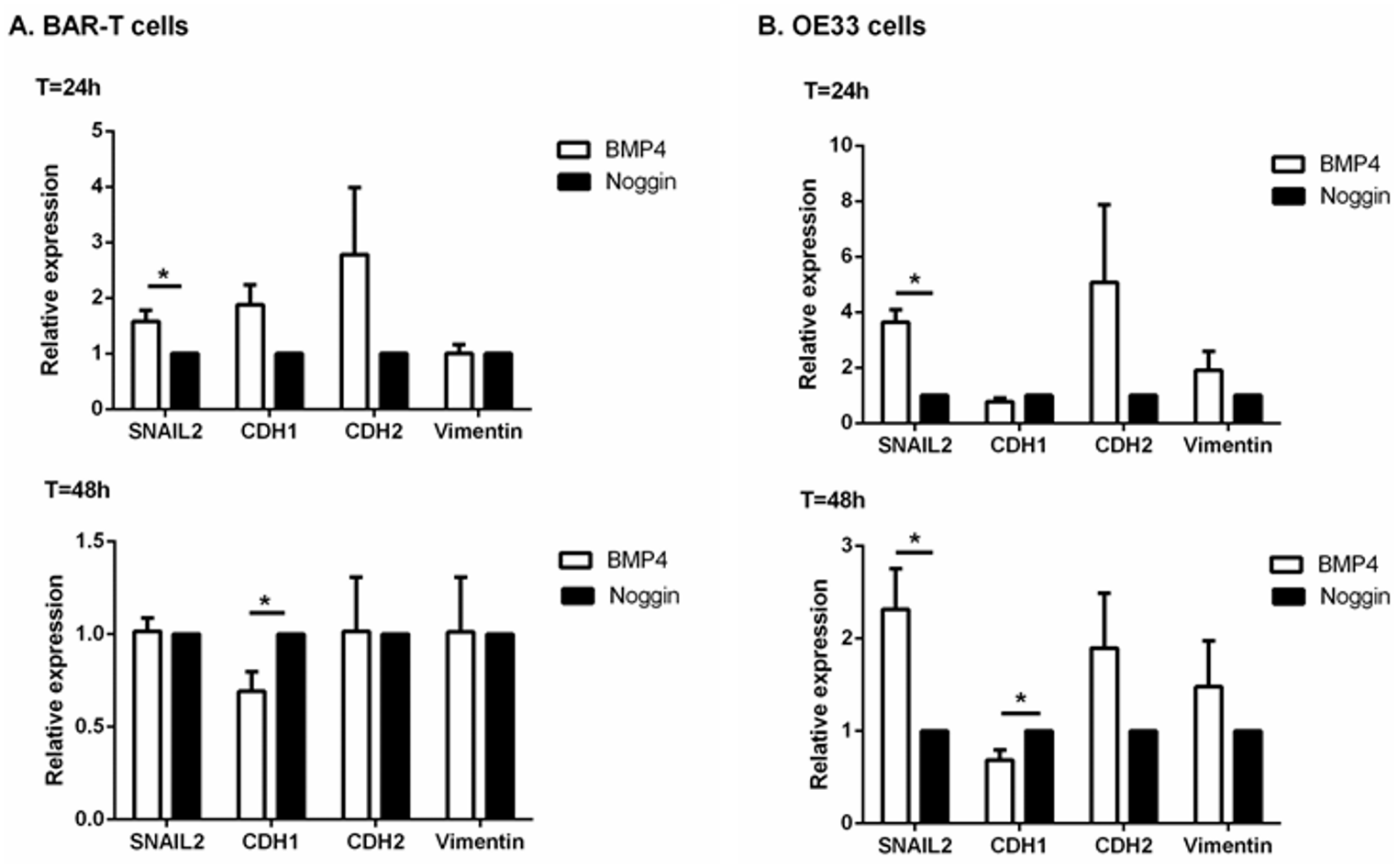
mRNA and protein expression of factors associated with EMT upon BMP4 or Noggin incubation. a. Q-RT-PCR was performed to determine mRNA expression of SNAIL2 and its target genes, CDH1, CDH2 and Vimentin. B2M was used for normalization. Data are relative to the mean ΔCt of cells incubated with Noggin and are expressed as mean±SEM. *p<0.05. b. Western blot analysis of BAR-T and OE33 cells showed that SNAIL2 expression was upregulated and CDH1 expression was downregulated in cells incubated with BMP4 compared to cells incubated with Noggin. Actin was used as loading control. Pictures are representative of three independent experiments.

EMT is an important contributor in regulating cell motility and to assess the consequences of BMP4 signaling in both BE and EAC, we investigated the ability of BMP4 signaling to induce EMT in BAR-T and OE33 cell lines. Q-RT-PCR was used to determine SNAIL2 expression, a transcription factor regulating EMT, in BAR-T and OE33 cells incubated with BMP4. BAR-T cells incubated with BMP4 for 24 hours showed a significant upregulation of SNAIL2 with a 1.6 (1.58±0.20; p = 0.02) fold induction compared to BAR-T cells incubated with Noggin. OE33 cells incubated with BMP4 showed a 3.7 (3.71±0.46; p = 0.02) fold induction of SNAIL2 after 24 hours and a 2.3 (2.31±0.44; p = 0.02) fold induction after 48h (Fig 4a). These results were confirmed by Western blot analysis, which showed upregulated SNAIL2 expression in BMP4 incubated BAR-T and OE33 cells (Fig 4b).

Next, we determined factors associated with EMT regulated by SNAIL2. CDH1, which is directly inhibited by SNAIL2, did not show a significant difference in mRNA expression in BAR-T (1.87±0.36) and OE33 cells (0.77±0.16) after 24 hours. However, after 48 hours, CDH1 expression was significantly (1.4 fold, 0.69±0.11; p = 0.02) downregulated in BAR-T cells incubated with BMP4 compared to cells incubated with Noggin. OE33 cells showed a tendency to CDH1 downregulation upon BMP4 incubation that was borderline significant (1.4 fold, 0.69±0.11; p = 0.06). Upon a one-tailed Wilcoxon test, CDH1 downregulation upon BMP4 incubation was statistically significant (p = 0.03). These results were confirmed by Western blot analysis, showing downregulation of CDH1 in cells incubated with BMP4 compared with cells incubated with Noggin after 48 and 72 hours (Fig 4b).

Mesenchymal markers, CDH2 and Vimentin did not show any significant difference after 24 and 48 hours. However, after 72 hours upon incubation with BMP4 in BAR-T cells, a strong tendency to upregulated mRNA expression of both CDH2 (7.85±7.89; p = 0.09) and Vimentin (4.68±6.78; p = 0.06) was seen. OE33 cells incubated with BMP4 showed also upregulation of mRNA of CDH2 (2.98±4.25) and Vimentin (2.27±2.92), although this was not statistically significant. We were not able to evaluate CDH2 and Vimentin protein expression in both BAR-T and OE33 cell culture experiments due to technical problems. As the positive control showed CDH2 and Vimentin expression, we concluded that it is likely that CDH2 and Vimentin expression in BAR-T and OE33 cells is very low.

## Discussion

BMP4 is known to play an important role in the development of BE in the esophagus. [9,13, 26] However, little is known about the expression and function of the BMP4 pathway in the neoplastic development towards EAC. In this study, we showed active BMP4 signaling in EAC biopsy specimens (Fig 1). We subsequently studied the functional role of BMP4 signaling in BAR-T and OE33 cell lines and found that BMP4 signaling decreased cell viability and increased cell migration (Fig 3). Furthermore, upregulated SNAIL2 expression was found in BAR-T and OE33 cells upon incubation with BMP4. CDH1, a known target of SNAIL2, was found to be downregulated. (Fig 4)

BMP4 is aberrantly expressed in many cancers. [7] In most cases there is an upregulated BMP4 expression compared with the corresponding normal tissue, for example in renal cell carcinoma, gastric cancer and squamous cell carcinoma originating in the head and neck region. [30–32] Previously, BMP4 expression was observed in both EAC and esophageal squamous cell carcinoma. [4] However, until now active BMP4 signaling was not confirmed in EAC. Here, we demonstrated expression of essential members of the BMP4 pathway in SQ, BE and EAC tissue. In addition we showed upregulation of BMP4, and, its downstream target ID2 in both BE and EAC tissue. Moreover more phosphorylation of SMAD1/5/8 was observed. This together with the fact that both cytoplasmic and nuclear SMAD4 staining was observed in BE and EAC suggests active BMP4 signaling in BE and EAC.

In epithelial neoplasms, cells are required to gain motile properties in order to metastasize, a process called EMT. Epithelial cells lose their cell-junctions and cell-extracellular matrix connections, reorganize their cytoskeleton and reprogram gene expression, which leads to a more invasive mesenchymal phenotype. EMT is not only important for cancer metastasis but also plays a major role in embryogenesis, wound healing and fibrosis. [33] The hallmarks of EMT are the downregulation of CDH1 and the upregulation of CDH2, Vimentin and α-smooth muscle actin. The process of EMT is controlled by transcription factors including SNAIL, TWIST and ZEB. [21] SNAIL2 is a transcriptional repressor and directly binds to the E2-homeobox promotor region of CDH1 leading to CDH1 downregulation. Additionally, through indirect mechanisms, SNAIL2 activates the expression of mesenchymal genes such as Vimentin and CDH2. [34]

The exact consequences of increased BMP4 signaling are not completely understood. However, the functional effects of active BMP4 signaling have been investigated *in vitro* in various cancer cell lines, showing the possibility of a tumor-specific reaction upon BMP4 activation. [7] For example, in pancreatic and ovarian cancer increased migration and decreased cell growth has been observed, while in brain tumors increased cell growth and in breast cancer reduced cell growth was demonstrated upon BMP4 incubation. [7] Recently, several studies have shown the ability of BMP4 to induce EMT or an EMT like response during embryogenesis [14,15], wound healing [16,17] and in various cancer cell lines [18–20]. *In vitro* cultured ovarian and pancreatic cancer cells showed upregulation of SNAIL2 and downregulation of CDH1 upon BMP4 incubation. [18,20] Gordon *et al*. also showed that BMP2, BMP4 and BMP7 were able to induce EMT in a pancreatic cancer cell line. [35] Our results showed that BMP4 is able to de-differentiate BE and EAC cells by upregulating SNAIL2 and subsequently downregulating CDH1 expression leading to a more invasive cell. In line with the results of Theriault *et al*. [18], the downregulation of CDH1 occurred after 48 hours of BMP4 incubation. Our results showed a tendency of CDH2 mRNA upregulation but we did not observe any effect on the expression of Vimentin. This may be explained by the fact that both CDH2 and Vimentin are no direct but indirect targets of SNAIL2 [21]. In addition, other transcription factors i.e. SNAIL1 or TWIST may also be important to completely induce EMT. Moreover, morphological changes take place concurrently with upregulated expression of mesenchymal markers and other studies observed morphological changes after 3–7 days. [17,18,20] Extending the experiments up to 72 hours, might induce morphological changes and upregulate the expression of mesenchymal markers in the esophagus. This is the first study focusing on the role of BMP4 signaling on an EMT like response and we believe that future studies are needed to fully elucidate this.

Previous studies investigating EMT in EAC have shown expression of the transcription factors SNAIL1, SNAIL2 and TWIST. [23] Rees *et al*. reported upregulation of mesenchymal genes, Vimentin and α-smooth muscle actin, and downregulation of CDH1 at the invasive margins of EAC compared to the central tumor using immunohistochemistry. [24] A study by Jethwa *et al*. concluded that SNAIL2 was upregulated in EAC compared to BE and SNAIL2 expression was inversely correlated with CDH1 expression. In addition, overexpression of SNAIL2 in OE33 cells resulted in downregulation of CDH1. [25] Previous studies have indicated that during malignant progression from BE to EAC, the expression of CDH1 is downregulated, which is known to be associated with a worse prognosis. [36–39] In addition to these studies, we showed that active BMP4 signaling resulted in downregulation of CDH1. In theory, active BMP4 signaling could be associated with a worse prognosis. In the future, manipulation of the BMP4 pathway may help to prevent neoplastic progression in BE patients.

In summary our study showed that the BMP4 signaling pathway, which is highly expressed and activated in BE and EAC, is capable of de-differentiating epithelial cells to an EMT like response in the esophagus through an upregulation of SNAIL2 and subsequently a downregulation of CDH1. In combination with the previous described literature, our study suggests that BMP4 signaling could induce malignant progression from BE to EAC. However, further studies are required to explore the precise molecular role of BMP4 activation in the esophagus.

## Supporting Information

S1 Fig Effect of BMP4 or Noggin incubation on cell viability. Cell viability assay of BAR-T (S1a Fig) and OE33 cells (S1b Fig) incubated for 24 and 48 hours with BMP4 or Noggin. Data are relative to control cells not incubated with BMP4 or Noggin and expressed as mean±SD. * p<0.05, ** p<0.01.
(DOCX)

## Abbreviations

BMP4: Bone Morphogenetic Protein 4
SQ: Squamous epithelium
BE: Barrett’s esophagus
EAC: esophageal adenocarcinoma
TGF-β: transforming growth factor β
CI: confidence interval
SD: standard deviation
SEM: standard error of the mean Q-RT-PCR, quantitative reverse transcriptase polymerase chain reaction
EMT: epithelial-mesenchymal transition
MET: mesenchymal-epithelial transition
