# Correction: BMP4 Signaling Is Able to Induce an Epithelial-Mesenchymal Transition-Like Phenotype in Barrett's Esophagus and Esophageal Adenocarcinoma through Induction of SNAIL2

**DOI:** 10.1371/journal.pone.0158755

**Published:** 2016-06-29

**Authors:** Christine Kestens, Peter D. Siersema, G. Johan A. Offerhaus, Jantine W. P. M. van Baal

There are panels missing from Figs 1 and 4. Please see the corrected Figs 1 and 4 here.

**Fig 1:**
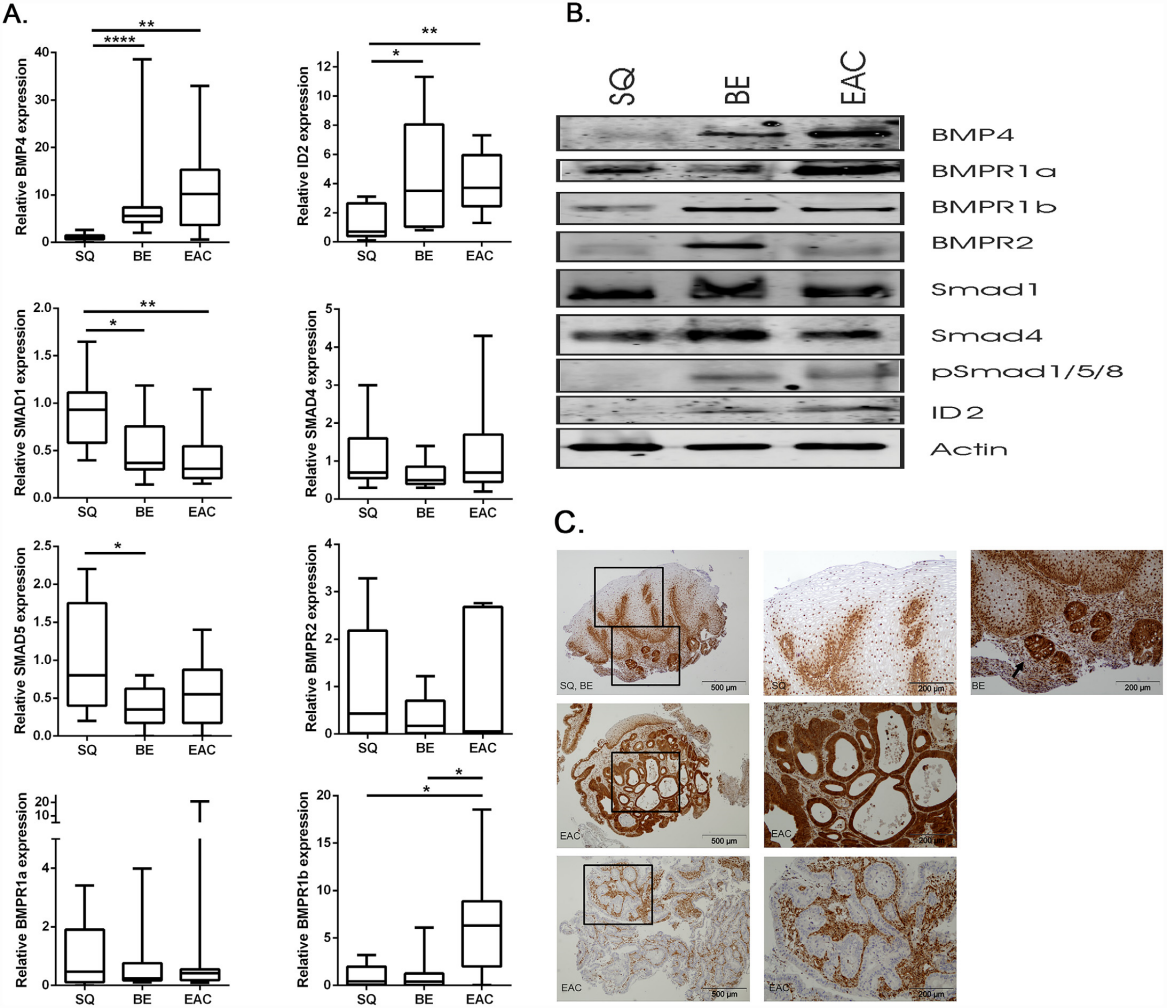
Expression of BMP4, BMP4 pathway associated molecules and the downstream target ID2. a. Q-RT-PCR was used to determine mRNA expression of BMP4, its downstream target, ID2, BMP4 associated receptors and SMAD molecules in SQ, BE and EAC biopsy specimens. B2M and GAPDH were used for normalization. Data are relative to the mean ΔCt of SQ biopsies and are expressed as box plots, representing the mean with the minimum and maximum values. *p<0.05, **p<0.01, ***p<0.001, ****p<0.0001. b. Western blot analysis in SQ, BE and EAC biopsy specimens showed BMP4, and ID2 expression and phosphorylation of SMAD1/5/8 in BE and EAC. Actin was used as loading control. Biopsy samples from 6 EAC patients were used. Representative pictures are shown. c. IHC showed nuclear and cytoplasmic expression of SMAD4 in 10 of 13 BE (arrowhead) and 11 out of 13 EAC tissue sections. EAC* represents a biopsy specimen with positive SMAD4 staining, EAC** represents a biopsy specimen with negative SMAD4 staining, stromal cells are SMAD4 positive and serve as internal control. Haematoxylin counterstain was used. Representative pictures are shown.

**Fig 4:**
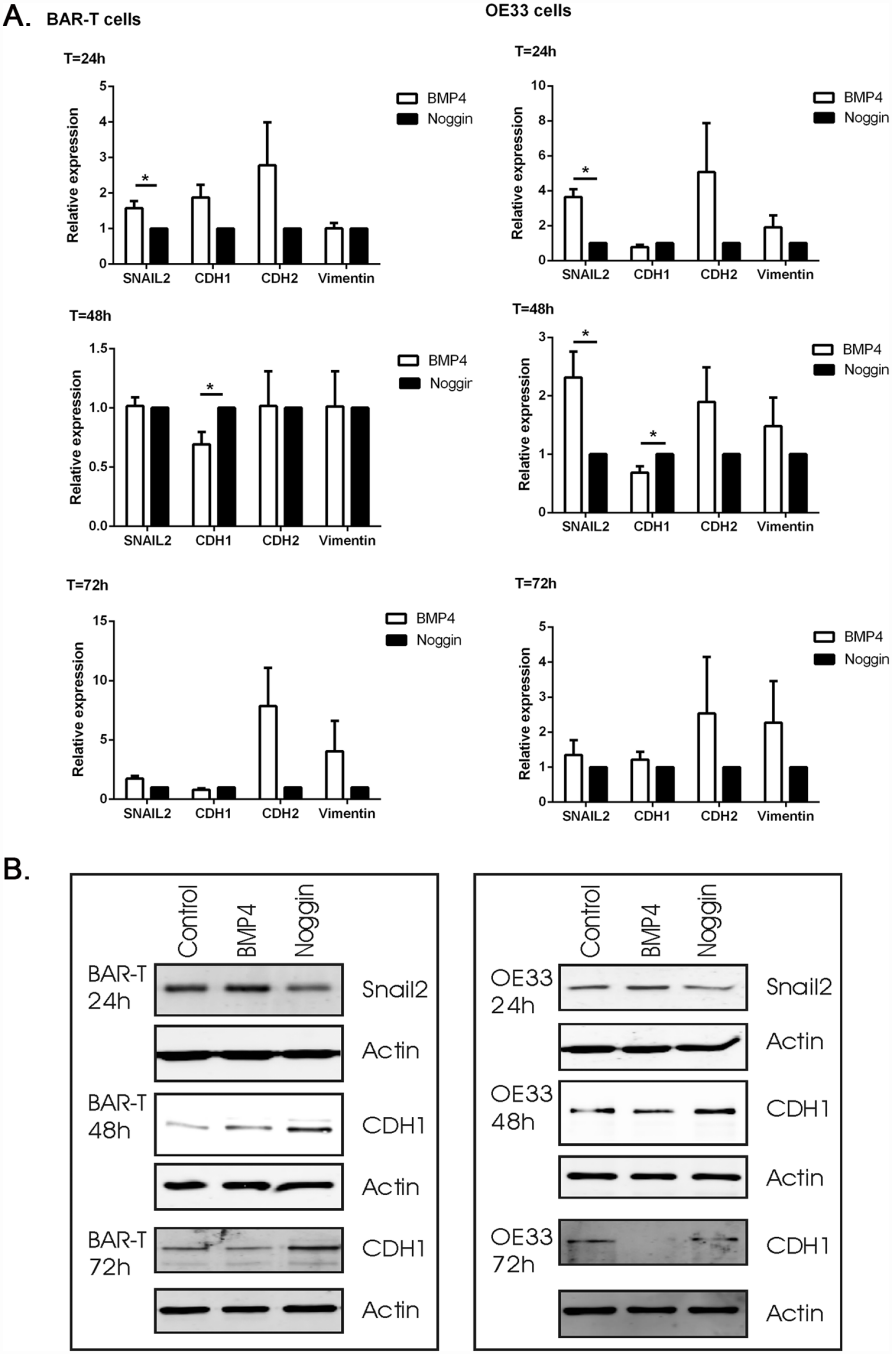
mRNA and protein expression of factors associated with EMT upon BMP4 or Noggin incubation. a. Q-RT-PCR was performed to determine mRNA expression of SNAIL2 and its target genes, CDH1, CDH2 and Vimentin. B2M was used for normalization. Data are relative to the mean ΔCt of cells incubated with Noggin and are expressed as mean±SEM. *p<0.05. b. Western blot analysis of BAR-T and OE33 cells showed that SNAIL2 expression was upregulated and CDH1 expression was downregulated in cells incubated with BMP4 compared to cells incubated with Noggin. Actin was used as loading control. Pictures are representative of three independent experiments.

## References

[1] Kestens C, Siersema PD, Offerhaus GJA, van Baal JWPM (2016) BMP4 Signaling Is Able to Induce an Epithelial-Mesenchymal Transition-Like Phenotype in Barrett’s Esophagus and Esophageal Adenocarcinoma through Induction of SNAIL2. PLoS ONE 11(5): e0155754 doi: 10.1371/journal.pone.0155754 27191723 10.1371/journal.pone.0155754 PMC4871520

