# Supplementary material for: BMP4 Signaling Is Able to Induce an Epithelial-Mesenchymal Transition-Like Phenotype in Barrett’s Esophagus and Esophageal Adenocarcinoma through Induction of SNAIL2
Source: PLoS One. 2016 May 18;11(5):e0155754. doi: 10.1371/journal.pone.0155754 (PMC4871520; doi:10.1371/journal.pone.0155754)
Supplement: S1 Fig — Cell viability assay of BAR-T (S1a Fig) and OE33 cells (S1b Fig) incubated for 24 and 48 hours with BMP4 or Noggin. Data are relative to control cells not incubated with BMP4 or Noggin and expressed as mean±SD. * p<0.05, ** p<0.01. (DOCX) [file pone.0155754.s001.docx]

**Supporting information**

**S1 figure. Effect of BMP4 or Noggin incubation on cell viability.**


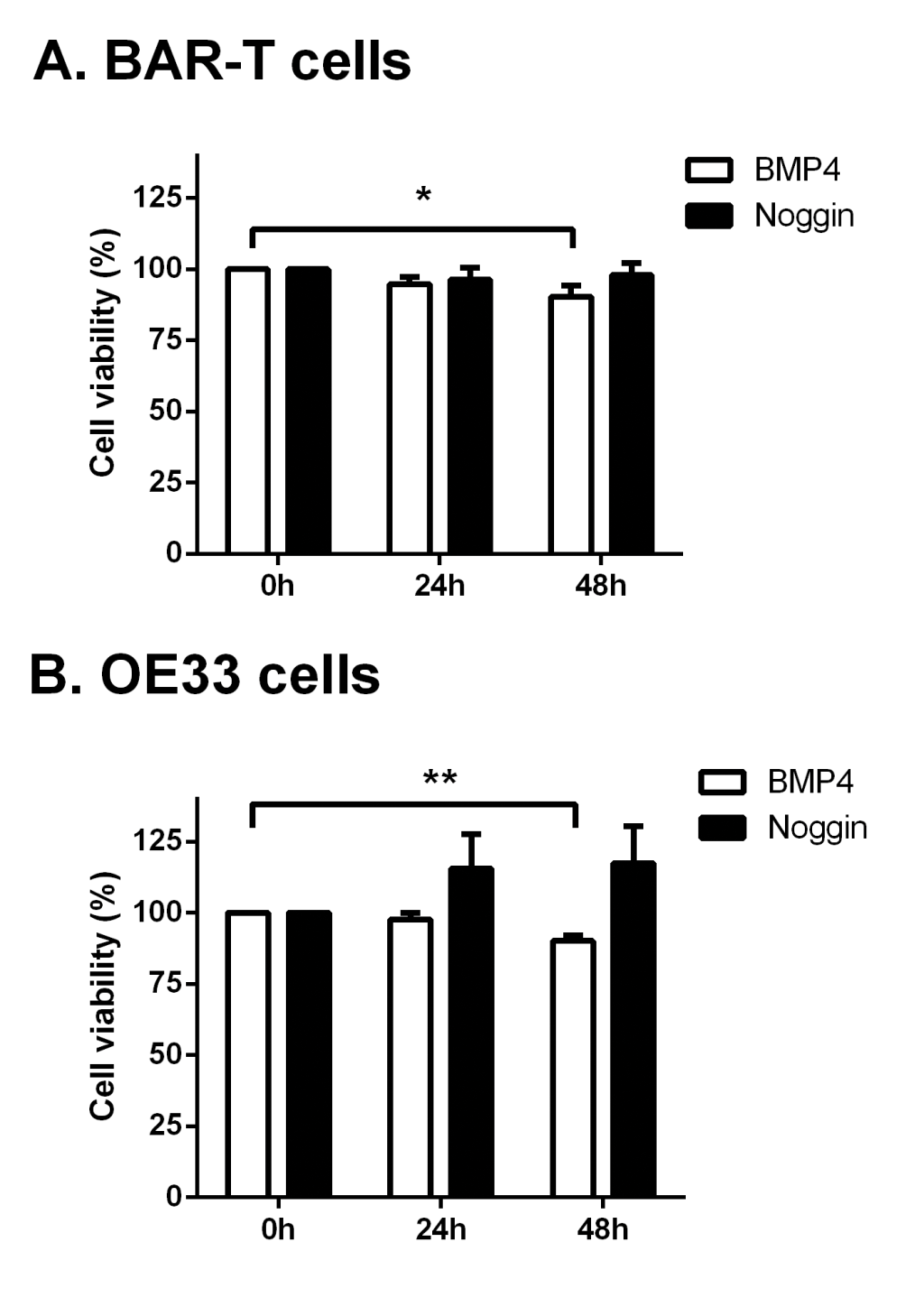


Cell viability assay of BAR-T (S1 Figure a) and OE33 cells (S1 figure b) incubated for 24 and 48 hours with BMP4 or Noggin. Data are relative to control cells not incubated with BMP4 or Noggin and expressed as mean±SD. * p<0.05, ** p<0.01.
